# AGE-FRIENDLY CARE AND MOBILITY IN HOSPITALIZED OLDER ADULTS WITH COGNITIVE IMPAIRMENT

**DOI:** 10.1093/geroni/igz038.1673

**Published:** 2019-11-08

**Authors:** Jasmine K Vickers, Richard E Kennedy, Shari Biswal, David James, Katrina Booth, Emily Simmons, Kellie Flood

**Affiliations:** 1 University of Alabama at Birmingham, Birmingham, Alabama, United States; 2 University of Alabama at Birmingham (UAB) Hospital, Birmingham, Alabama, United States

## Abstract

Hospitalization of older adults with cognitive impairment (CI) has been associated with higher risk for adverse outcomes. Acute Care for Elders (ACE) Units were developed to meet the unique hospital care needs of older adults and have been associated with reductions in functional decline and readmissions. The Virtual ACE intervention was developed to disseminate ACE principles across hospital units. Virtual ACE included training interprofessional providers to utilize screens and care protocols to optimize care for older adults on eight units at a large academic medical center. We conducted a preliminary analysis of mobility and patient outcomes before and after Virtual ACE among 192 older adults with CI on hospital admission. Chi-Square tests were used to examine the associations between Virtual ACE and patient outcomes. There were statistically significant pre vs. post improvements in patients’ mobility from bed to chair (30% vs. 51%, p=0.011) and on the unit hallway (12% vs. 27%, p=0.046). Although not statistically significant, there were also improvements in hospital room mobility (39% vs. 50%, p=0.214) and documentation of activities of daily living (ADL) screens (70% vs. 80%, p=0.196). There were non-significant reductions in pressure ulcer prevalence (26% vs. 22%) and restraint use (5% vs. 0%) during the hospital stay. Pain was similar before and after Virtual ACE. Virtual ACE was associated with increased mobility and slight reductions in adverse outcomes. As increased hospital mobility improves patient functioning post-discharge, Virtual ACE has the potential to maintain function and enhance outcomes in hospitalized older adults with CI.

